# Access to transition-related health care among transmasculine people in India: A mixed-methods investigation

**DOI:** 10.1371/journal.pgph.0003506

**Published:** 2024-10-29

**Authors:** Venkatesan Chakrapani, Heather Santos, Madhusudana Battala, Shaman Gupta, Satvik Sharma, Aditya Batavia, Sahil Jamal Siddiqui, Kelly A. Courts, Ayden I. Scheim

**Affiliations:** 1 Centre for Sexuality and Health Research and Policy (C-SHaRP), Chennai, India; 2 National Institute of Advanced Studies, Bengaluru, India; 3 Department of Epidemiology and Biostatistics, Dornsife School of Public Health, Drexel University, Philadelphia, PA, United States of America; 4 Population Council, New Delhi, India; 5 Our Health Matters, Dehradun, India; 6 Our Health Matters, New Delhi, India; 7 TWEET Foundation, Bangalore, India; 8 Transmen Collective, New Delhi, India; 9 Department of Epidemiology and Biostatistics, Schulich School of Medicine and Dentistry, Western University, London, Canada; 10 Li Ka Shing Knowledge Institute, Unity Health Toronto, Toronto, Canada; Centre for Women’s Development Studies, INDIA

## Abstract

Little research has examined the health care experiences of transmasculine people in India, where government initiatives to improve access to transition-related (also called gender-affirmative) care have recently been announced. We draw on data from **‘**Our Health Matters’, a mixed-methods community-based participatory research project, to characterize the transition-related care experiences of transmasculine people in India. Peer researchers conducted 40 virtual qualitative interviews in Hindi or Marathi from July to September 2021. Between November 2022 and January 2023, 377 transmasculine people participated in a multi-mode survey available in five languages. Qualitative data were analysed with a combination of framework analysis and grounded theory techniques. Data were mixed using a convergent parallel approach. Transmasculine persons’ care journeys began with information-seeking, relying on peers and internet searches. In choosing between the public and private healthcare systems, they weighed issues of quality and affordability: the public system was perceived as lower-quality and difficult to access but most could not afford private care, leading to delays in care. Indeed, unmet need was common; 36.4% of survey participants were planning but had not begun to receive transition-related care and 80.2% wanted at least one transition-related surgery. Although some participants encountered stigma and refusal of care when seeking hormones, survey participants reported largely positive experiences with their hormone prescribers, which may reflect the influence of peer referrals. Participants underwent psychological assessments prior to transition-related care, which some experienced as disempowering and a barrier to disclosing mental health challenges. Finally, participants who were able to access care reported improved well-being, although surgical dissatisfaction was not uncommon (26.2%). Trans-inclusive medical training and continuing education are critical to enhancing access to high-quality transition-related care. Transmasculine people generally relied on peers and grassroots organizations for information, system navigation, and financial assistance. Strengthening these existing community resources may improve access to care.

## Introduction

Transmasculine people in India are increasingly visible in the public consciousness and government policies. The 2011 census provided an estimate of at least 0.48 million transgender (trans) people in India, although it did not provide an estimate for transmasculine people as the term “Other” was used and the option of choosing male or female was provided to all [1]. In this article, the term “transmasculine people” refers to those who were assigned a female gender at birth and who identify as transgender, transmasculine, trans man or man. Gender affirmation is a critical determinant of trans health and refers to internal and external recognition of one’s gender, encompassing psychological (self), social (e.g., use of proper names and pronouns), legal (match between self-affirmed gender and gender noted on legal identity documents), and medical dimensions [2]. In this paper, we refer to medical gender affirmation as transition-related care to reflect community language use and for conceptual clarity (as medical interventions do not affirm or disaffirm gender identity). Transition-related care can include hormone therapy and surgeries, which are considered essential health services for trans people who need them, as they assist in changing the body in congruence with self-affirmed gender identity [3, 4]. Specifically, testosterone and chest masculinization surgery are the treatments most desired and accessed by transmasculine people [5]; many also need hysterectomy and/or oophorectomy, genital reconstruction (metaoidioplasty or phalloplasty), or other surgeries (e.g., facial masculinization). Transition-related care is associated with a reduction in gender dysphoria (distress related to gender incongruence) and an improvement in mental health and quality of life [6, 7].

There is wide variation in access to transition-related care globally, ranging from this care being considered a human right in some countries, to trans and gender non-conforming identities being criminalized in others [8, 9]. In India, the recently enacted Transgender Persons (Protection of Rights) Act, 2019 and its associated 2020 Rules explicitly recognize the need to provide “sex reassignment surgery” and hormone therapy for trans people and direct the government to provide such services within the public healthcare system [10]. Despite the passage of this law, trans persons still struggle to access health services due to discrimination and inaccessibility of health facilities across the country [11]. Many states also do not provide comprehensive insurance coverage as required by the Act [11]. The Act also recommended that the government should prepare a “health manual related to sex reassignment surgery” in line with the World Professional Association for Transgender Health (WPATH) guidelines [3]. However, no such government-issued national guidelines are available. Guidance documents have been released by Indian non-governmental agencies such as the Association for Transgender Health in India [12, 13] and Sappho for Equality [14], but have not been endorsed by the Indian government [11].

India’s central government has introduced the ’Ayushman Bharat TG Plus’ card, providing trans persons with health insurance coverage of INR 5 lakhs (~USD 5800) per year and access to over 50 health services, including gender-affirmative surgeries [15]. The SMILE scheme launched in 2022 aims to provide comprehensive rehabilitation and healthcare for trans persons, including coverage for gender-affirmative surgeries [16]. In addition, the Tamil Nadu state government provides free gender-affirmative surgeries in at least three government hospitals and the Kerala state government has recently announced plans to roll out "queer-friendly hospital initiatives" [17]. However, implementation challenges remain, as facilities for gender-affirmative healthcare are still limited in many government hospitals across India.

The barriers faced by Indian transfeminine people in accessing HIV-related healthcare services [18, 19] as well as transition-related care [20] have been documented. However, recent scoping reviews have documented a near-total lack of peer-reviewed research on access to gender transition services for transmasculine people in India, as well as in other low-income and middle-income countries [21, 22]. A 2024 systematic review on barriers to accessing gender-affirming surgeries included 25 articles; none from India [23]. This article addresses this gap in the literature by exploring transmasculine people’s access to and use of transition-related services—particularly hormone therapy and surgeries. The study findings are expected to inform health policies and programs for medically necessary gender transition services for transmasculine people in India.

## Methods

### Data source

Our Health Matters: Indian Trans Men and Transmasculine Health Study was a community-based participatory mixed-methods study of transmasculine people’s mental health and access to health care in India. The study partnership began in 2017, when Indian trans community leaders engaged the Principal Investigator to explore research opportunities. The project’s research questions and methods were informed by a formative research phase, including community consultations with transmasculine people in New Delhi, Mumbai, and Bengaluru in 2018 and participatory planning meetings with the study team. The study was led by a Steering Committee of Indian transmasculine people and academic researchers (trans and cisgender) from India, Canada, and the United States, and conducted in partnership with two grassroots transmasculine-led organizations. Using a “community control” model of community-based participatory research [24], the Steering Committee had decision-making authority at all stages of the project. Additional information about the study, including a topline report on the survey in English and Hindi, and qualitative research reports in Hindi, Marathi, and English, can be found at www.ourhealthmatters.in. Preliminary qualitative findings regarding access to transition-related health care were published in one of the online reports [25]. Institutional Review Boards at Drexel University, the Population Council, and the Centre for Sexuality and Health Research and Policy approved the study.

## Data collection

Using an exploratory sequential (qual → QUANT) study design, data were collected in two phases: in-depth qualitative interviews were conducted from 7 July to 12 September 2021 and quantitative survey data were collected from 2 November 2022 to 24 January 2023. All study participants were trans men or transmasculine persons (i.e., were assigned female at birth and identified as men or transmasculine) aged 18 or above. The study was originally designed to combine in-person and online data collection in two urban areas (Delhi and Mumbai), in the primary local languages (Hindi and Marathi). In response to the COVID-19 pandemic, the first phase was conducted virtually. As a result, eligibility was extended to persons living anywhere in India who could participate in one of the study languages. Participants did not need to have undergone any transition-related care.

### Qualitative interviews

Recruitment and data collection for qualitative interviews took place online or by telephone due to COVID-19-related public health measures. Three research assistants from the transmasculine community (native speakers of Hindi and Marathi and conversant in English) were hired and trained to conduct recruitment and in-depth qualitative interviews. Specifically, research assistants participated in an interactive three-day training facilitated by experienced qualitative researchers, conducted practice interviews, and received weekly refresher sessions.

Participants were recruited through community outreach, including fliers circulated within transmasculine community networks, regional and national social networking groups, and social media. Study materials were available in English, Hindi, and Marathi. Potential participants contacted research staff to express interest, at which point they were screened for eligibility and asked basic demographic questions. This information was used to purposively select 40 participants who were diverse with respect to age, geographic location, and socioeconomic status.

A semi-structured interview guide included questions about family experiences, social and community support, experiences of discrimination, mental health, and access to health care. Interviews lasted about 45 minutes to 1 hour, were conducted in Hindi or Marathi, and were audio-recorded, transcribed verbatim, and translated into English for analysis. Participants provided written informed consent and received an honorarium of INR 500 via electronic funds transfer.

### Quantitative survey

The *Our Health Matters* questionnaire was programmed in Qualtrics and available online for self-administration and in-person on electronic tablets with transmasculine peer research assistants in four geographic areas (Delhi, Mumbai, Bengaluru, West Bengal). These locations were selected based on both the candidate pool and the team’s desire to ensure representation of the southern and eastern states in India, in addition to the two largest urban areas in the country. Research assistants were primarily responsible for survey promotion but also administered surveys on electronic tablets if participants needed or wanted assistance. They participated in a three-day in-person training and weekly supervision sessions. Survey participants were recruited using similar online outreach approaches as in the qualitative phase, with the addition of face-to-face outreach by research assistants at trans and LGBT community events, as well as study-specific events organized by team members in Assam (Dibrugarh and Guwahati), Bengaluru, and Hyderabad. The target sample size was 300, based on power calculations for the primary study outcome (depressive symptoms).

The questionnaire was divided into sections addressing demographics, access to legal gender recognition and government welfare programs, experiences of discrimination and acceptance, social supports, mental health status, and access to health care. The order of sections was randomized to prevent disproportionate levels of missing data due to failure complete the survey (break-off). Questions about health care access were either adapted from previous trans health studies [26, 27] or developed *de novo* based on preliminary findings from the qualitative interviews. The questionnaire was developed in English, translated to Hindi and Marathi, and then back-translated to ensure conceptual equivalence. Discrepancies were resolved through discussion between study investigators, translators, and transmasculine native speakers. The questionnaire was pilot tested by transmasculine Steering Committee members and research assistants and revised for clarity and to reduce length; the final version took approximately 30 minutes to complete (via self-administration). Based on community requests, the questionnaire was also translated to Bengali and Telugu shortly after data collection launched; these versions were not back-translated but were reviewed by bilingual transmasculine research assistants.

After reviewing the information letter programmed into the questionnaire, participants indicated their consent by marking a checkbox. After completing the questionnaire, participants could opt-in to provide contact information for receipt of an INR 500 honorarium via electronic funds transfer. Following best practices for online surveys, we used multiple strategies to prevent and identify fraud, including targeted recruitment, eligibility screening questions (prior to disclosure of the incentive amount), CAPTCHA, attention and consistency checks, review of write-in responses, verification of geolocation in India using IP addresses, and automated Qualtrics tools to detect duplicate or “bot” responses [28]. When necessary, we contacted participants to verify their identities by confirming answers to questions in the compensation form (e.g., name, favorite color). We excluded data from participants who could not be verified, and those who did not complete at least two of five survey sections.

### Data analysis

Authors MB and AIS developed an initial codebook based on domains of the qualitative interview guide. Five team members including MB, AIS, VC, and HS coded the first five transcripts in duplicate; this process was used to iteratively refine the codebook and to reach consensus on application of codes. Thereafter, each transcript was coded by one of two trained graduate research assistants, under AIS’ supervision. The codebook contained both ‘a priori’ codes (e.g., “gender dysphoria diagnosis”, “starting hormone therapy”) derived from the topic guide (consistent with the ‘framework analysis’ approach used in policy-oriented research) [29], as well as ‘emergent codes’ (e.g., before and after Transgender Persons [Protection of Rights] Act of 2019, problems with changing legal gender identity) that were identified as we analysed the data, consistent with the techniques of the grounded theory approach [30]. The combination of these two coding approaches (called Tabula Geminus or ‘both/and’ approach) is compatible and explained in the methodology literature [31, 32]. ATLAS.ti software [33] was used for data management and coding. We shared the preliminary findings with transmasculine Steering Committee members to elicit their feedback and incorporated their comments, a form of ‘respondent validation’ or ‘member checking’ [34, 35].

Although the overall study used an exploratory sequential mixed-methods design (using interview data to develop the questionnaire), in this paper we employ a convergent parallel analytic approach [36] by comparing and contrasting qualitative findings to descriptive statistics (frequencies) from the quantitative survey and to derive inferences from both qualitative and quantitative data (complementarity). In presenting results, we distinguish between qualitative in-depth interview (“IDI”) participants and survey participants. IDI participants are referred to using unique identifiers; HI and MI refer to interviews conducted by Hindi- and Marathi-speaking research staff respectively (this largely corresponds to language of interview; however, some interviews were multilingual).

## Results

### Participants

IDI participants ranged in age from 20 to 50 (Mean = 27.6, SD = 6.7) and lived in 10 Indian states: Assam (n = 1), Delhi (n = 5), Jharkhand (n = 1), Haryana (n = 2), Karnataka (n = 1), Maharashtra (n = 24), Rajasthan (n = 2), Tamil Nadu (n = 1), Telangana (n = 1), and Uttar Pradesh (n = 2). Nineteen IDIs were conducted by Hindi-speaking interviewers and 21 by Marathi-speaking interviewers. One-quarter (n = 10) of participants reported no source of employment while 50% (n = 20) were employed in the private sector, 15% had another form of employment (e.g., internship, self-employment), 7.5% were students (n = 3), and one was retired. Survey participants lived in 22 Indian states and union territories, with the largest numbers of participants in Haryana (n = 51), Delhi (n = 50), Uttar Pradesh (n = 44), West Bengal (n = 39), Maharashtra (n = 34), and Karnataka (n = 23). [37] Additional characteristics of survey participants are shown in Table 1. Most completed the survey in English (70.0%), were between 18–24 (49.6%) years or 25–34 (41.1%) years old and had a post-secondary education (55.4%). Over one-third (36.4%) were planning to receive transition-related care but had not begun to do so, while 28.4% were in process of transition-related care and 24.3% said they had completed all needed care.

**Table 1:** Demographics of Our Health Matters survey participants (n = 377).

Characteristic	% (n)
Survey completion language	
English	70.0% (264)
Hindi	23.3% (88)
Marathi	2.7% (10)
Bengali or Telugu	4.0% (15)
Age category	
18–24	49.6% (187)
25–34	41.1% (155)
35–46	9.3% (35)
Highest level of education (n = 372)	
Primary (5th grade)	4.3% (16)
Secondary (10th grade)	6.2% (23)
Higher secondary school (12th grade)	34.1% (127)
University degree or diploma	55.4% (206)
Occupation (n = 370)	
Unemployed	28.9% (107)
Student	28.9% (107)
Daily wage labourer	6.8% (25)
Employee	24.9% (92)
Self-employed	7.0% (26)
Other	3.5% (13)
Caste (n = 370)	
General Category	47.8% (177)
Scheduled Caste/Scheduled Tribe	26.2% (97)
Scheduled Tribe	10.0% (37)
Other Backward Class	13.8% (51)
Do not want to answer/ Not a member of any caste	12.2% (45)
Religion (n = 372)	
Buddhist	2.7% (10)
Christian	4.8% (18)
Hindu	78.8% (293)
Jain	0.8% (3)
Muslim	7.3% (27)
Sikh	3.2% (12)
Would rather not say	2.4% (9)
Identifies as a disabled person or a person with a disability (n = 375)	10.7% (40)
Self-identified sexual orientation (n = 363)	
Asexual	4.5% (15)
Gay, bisexual, pansexual, or queer	15.4% (51)
Lesbian	6.6% (22)
Not sure, questioning, other	16.7% (55)
Straight or Heterosexual	56.8% (188)
Lived gender in day-to-day life (n = 361)	
Man or boy	58.2% (210)
Woman or girl	8.3% (30)
Sometimes man/boy, sometimes woman/girl	27.1% (98)
Non-binary	6.4% (23)
Average monthly personal income in INR (n = 285)	
0 to 1,000	42.5% (121)
1,001 to 10,000	30.5% (87)
10,001 to 20,000	16.5% (47)
More than 20,000	10.5% (30)
Transition-related care status (n = 341)	
Has had all care they need/want	24.3% (83)
In the process	28.4% (97)
Planning but not begun	36.4% (124)
Not planning	5.9% (20)
Not sure about seeking care	5.0% (17)

### Overview of qualitative findings

**Fig 1:**
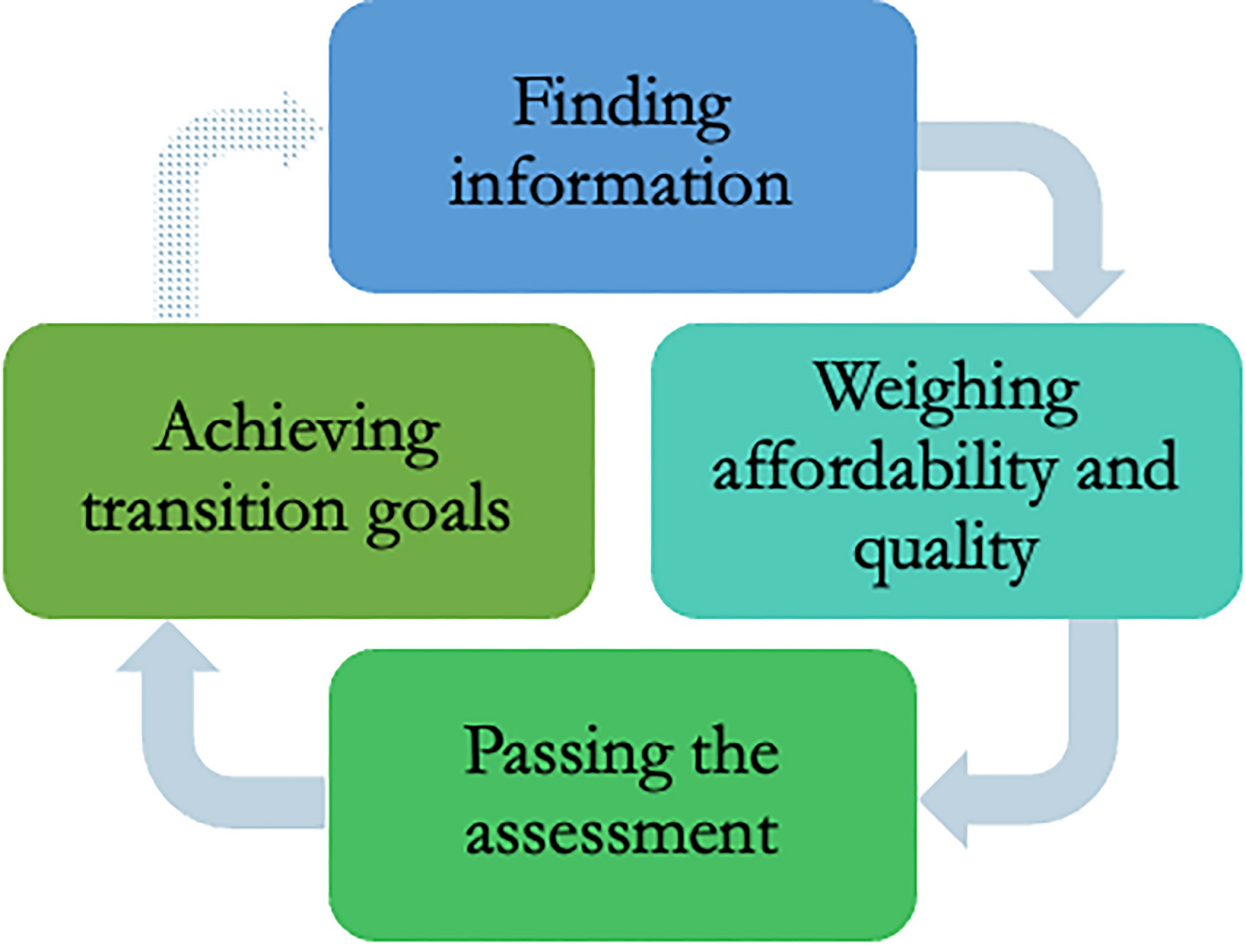
Representation of the process of seeking and accessing transition-related health care for transmasculine participants. The dotted arrow indicates the potentially cyclical nature of accessing gender-affirmative care. For example, those who have already accessed one type of procedure (e.g., hormone therapy), may then seek information on another (e.g., chest surgery). *Adapted from [25]*.

We found considerable diversity in the experience of transmasculine participants in accessing and using transition-related care. As shown in Fig 1, in general, participants began with a search for information on medical treatments and trans-friendly, experienced doctors, mainly through their peers and the Internet. They weighed considerations of affordability and quality of the limited number of available services and navigated the approval process for transition-related care, which usually involved assessment by one or two mental health professionals before initiation of hormone therapy. Along the way, participants described negative and positive experiences with doctors, nurses, and other healthcare providers. Finally, despite several challenges, participants who had achieved their transition-related goals described the positive impacts of transition-related care on their health and well-being, and those who could not afford it discussed plans to achieve their goals. Additional participant quotes related to each stage of the gender-affirming care journey are provided in (S1 Table).

### Finding information about transition-related procedures and services

IDI participants generally received information about transition-related care from transmasculine peers they knew online or offline (often introduced through support groups). Older participants noted that younger community members would come to them for transition-related information, as they had more experience accessing care. A participant shared how a peer helped him: “One friend on Facebook is a trans man. He started the [transition] process in Mumbai but did the surgery in Thailand…I got to know that [transition-related surgery] can happen in [hospital name].” (26, Kalyan, Maharashtra, MI12)

Some IDI participants conducted internet searches to identify potential trans-friendly doctors and transition-related procedures. This was common across age groups; however, participants who began their transitions earlier noted that information was less readily available when they first needed it, although information is now available online and through social media. As a participant described: “The hardest part is that at the time of the transition, I was very young… When it was 2012 or 2014, there was not much information on Instagram or Facebook.” (23, Mumbai, MI25). Consistent with these qualitative findings, of survey participants who had ever been on hormone therapy, 77.8% said they learned about the doctor(s) who had provided their hormones through a trans friend and 32.5% learned of their doctor(s) via an internet search; only 3.6% were referred to a hormone prescriber by a family doctor (Table 2).

**Table 2:** Health care experiences of Our Health Matters survey participants.

Characteristic	% (n)
Has a regular doctor for general health issues (n = 347)	
No	46.4% (161)
Yes	53.6% (186)
Comfort discussing trans or non-binary health issues with regular doctor (n = 158)^1^	
Mostly or very comfortable	54.4% (86)
Somewhat or not at all comfortable	45.6% (72)
Regular doctor is knowledgeable about trans or non-binary issues (n = 157)^1^	
Mostly or very knowledgeable	63.7% (100)
Somewhat or not at all knowledgeable	36.3% (57)
Referral source to doctor who provided hormones* (n = 194)^2^	
I nternet search	32.5% (63)
Family doctor	3.6% (7)
Trans friend	77.8% (151)
Non-trans friend	2.6% (5)
Ever taken hormones (testosterone) (n = 344)	
No	43.6% (150)
Yes	56.4% (194)
Currently taking hormones (testosterone) (n = 342)	
No	47.7% (163)
Yes	52.3% (179)
Had any gender-affirmative surgery (n = 341)	
No	74.5% (254)
Yes	25.5% (87)
Mastectomy or chest reconstruction (’top surgery’) (n = 334)	
Don’t want to have	8.1% (27)
Want to have	68.6% (229)
Have had	23.4% (78)
Hysterectomy (removal of uterus) or Oophorectomy (removal of ovaries) (n = 334)	
Don’t want to have	9.3% (31)
Want to have	80.2% (268)
Have had	10.5% (35)
Metoidioplasty or Phalloplasty (’bottom surgery’) (n = 323)	
Don’t want to have	17.0% (55)
Want to have	79.9% (258)
Have had	3.1% (10)
Other surgery (n = 205)	
Don’t want to have	31.4% (69)
Want to have	59.1% (130)
Have had	2.7% (6)
Paid for surgery with:* (n = 87)^3^	
Own savings	47.1% (41)
Money from family	29.9% (26)
Money from friends	14.9% (13)
Crowd funding donations	10.3 (9)
Support from workplace	4 .6%(4)
Using a government hospital	1.1% (1)
Visited a psychiatrist for a gender dysphoria (GD) letter (n = 336)	
No	32.7% (110)
Yes	67.3% (226)
Had a negative experience when visiting psychiatrist for GD letter (n = 226)^4^	
No	85.4% (193)
Yes	14.6% (33)
Negative experience included:*	
Used transphobic or disrespectful language	4.9% (11)
Asked inappropriate or invasive questions	7.5% (17)
Tried to convince you not to medically transition	4.4% (10)
Refused to provide a GD letter	4.0% (9)
Had a positive experience when visiting psychiatrist for GD letter (n = 226)^4^	
No	11.9% (27)
Yes	88.1% (199)
Positive experience included:*	
Used trans-friendly and respectful language	78.8% (178)
Supported decision to medically transition	31.4% (71)
Comfort discussing trans or non-binary health issues with endocrinologist (n = 169)	
Mostly or very comfortable	89.9% (152)
Somewhat or not at all comfortable	10.1% (17)
Endocrinologist is knowledgeable about trans or non-binary issues (n = 167)	
Mostly or very knowledgeable	91.0% (152)
Somewhat or not at all knowledgeable	9.0% (15)
First took hormones without a doctor’s prescription (n = 192)^2^	
No	82.3% (158)
Yes	17.7% (34)
Doctor has denied request for a prescription for hormones (N = 191)^2^	
No	80.6% (154)
Yes	19.4% (37)
Takes hormones by injection (n = 180)^2^	
No	2.2% (4)
Yes	97.8% (176)
Has had any surgery done in India (n = 85)^3^	
No	9.4% (8)
Yes	90.6% (77)
Overall satisfaction with surgery/ies (n = 84)^3^	
Very dissatisfied	13.1% (11)
Dissatisfied	13.1% (11)
Neither satisfied nor dissatisfied	19.0% (16)
Satisfied	34.5% (29)
Very satisfied	20.2% (17)

Among participants who indicated having a general doctor

Among participants who had ever taken hormones

Among participants who have had surgery

Among participants who visited a psychiatrist for a GD letter

*Question was check-all-that-apply and percentages do not add up to 100%

Reliance on word-of-mouth and the Internet introduced challenges for participants who needed to determine whether the information was authentic and reliable, since no official medical or community-vetted sources were available. Information from peers was diverse and conflicting, leaving an IDI participant unsure of which peer’s recommendation to follow: “I met a lot of [trans] people on social media. They would suggest to me to go to this doctor and go to that doctor.” (28, Mumbai, MI16)

A lack of adequate and correct information about transition-related care and trans-friendly services delayed the initiation of care and led to a waste of money and time as participants desperately tried to locate trans-friendly services. One IDI participant complained:

The only problem then [2016] was that we did not know where transition care was provided. I was searching but did not get proper information. When I started taking hormones, I realized there are many more people like me. I spent a lot of money. I took time from my college schedule and wandered a lot for this. (23, Mumbai, MI25)

A few participants were guided by trans-friendly doctors regarding the current practice in initiating hormone therapy, which required obtaining a letter from a psychiatrist:

“He [a trans-friendly doctor] told me that the journey starts with a psychiatrist from whom I will get a GID letter. I had to talk to the [psychiatrist] about myself, then I would get the Gender Identity Dysphoria letter. After which I would be ready for [hormone therapy] by going to an endocrinologist. (40, Nagpur, Maharashtra, HI14)

### Weighing the affordability of services

The affordability of transition-related care–testosterone injections and gender-affirmative surgeries–was a crucial factor in access to care. Although we did not explicitly ask, none of the IDI participants mentioned having government or private health insurance. Survey participants were mostly unemployed or students, with low incomes overall; of those who provided income data, 89.5% made 20,000 INR or less per month (20,000 INR is approximately 240 USD as of September 2023) (Table 1). IDI participants noted having to delay transitioning until they felt financially secure and independent. For example, a 26-year-old in Maharashtra shared [MH12]: “In March 2019, I was unemployed, so I stopped taking injections. I didn’t want to take money from my family, so I stopped taking injections. After 5 to 6 months, I again started taking injections.” Some participants sought assistance from community-based organizations to fund their hormone treatment:

I was given an injection [anabolic steroid for muscle-building] that I had to buy every month. It was costly. So, I stopped taking it and started taking [regular testosterone injection] hormones… [Organization name] helped me with 3,000 Rupees. (25, Washim, Maharashtra, MI15).

The lack of trans-friendly doctors to prescribe hormones and high consultation fees forced some participants to buy hormones from over-the-counter pharmacies or the Internet. Of the 56.4% of survey participants who had ever taken hormones, 19.4% had ever been denied a prescription for hormones and 17.7% began hormone therapy without a prescription (Table 2). An IDI participant shared (26, Chennai, Tamil Nadu, HI22): “I ordered the [hormone] injection from an online pharmacy, which is delivered at home. Then, I take it at home.” Although self-injection was uncommon overall (34.7% [61/176] of survey participants on hormone therapy reported self-injecting), negative experiences in clinic settings compelled some IDI participants to self-inject hormones. One participant explained what happened after he asked a nurse in a private clinic to give him a hormone injection:

The nurse looked at me from top to bottom. The second time I was seen by a male doctor who asked many strange questions. He asked me to show my prescription and then gave me an injection…I was wondering what they would say next…Then I learned to give injections myself. (28, Mumbai, MI16)

Few participants had been able to access transition-related surgery. As shown in Table 2, one-quarter (25.5%) of survey participants had completed any type of surgery, most often chest surgery (23.4%), while the vast majority said they wanted to have surgery but had not yet done so (ranging from 68.6% for chest surgery to 80.2% for hysterectomy).

In general, transition-related surgical services were scarce, particularly within the public healthcare system (government hospitals). Where gender-affirmative surgeries were performed in government hospitals, delays and perceived poor quality of surgeries (based on peers’ or own experiences) led some to go to private hospitals. As an IDI participant reported: “We decided to go to a government hospital as care in private hospitals was costly…We went there for four to five months to get a date for the surgery…But it did not work out. Then I saved some money and went to a private hospital.” (36, Pune, Maharashtra, MI26). Notably, survey participants were asked how they financed gender-affirmative surgeries and only one respondent reported having had surgery in a government hospital.

Based on peer recommendations and Internet searches, participants believed that care in some private hospitals was of higher quality, with shorter wait times. However, costs were unaffordable, leading to postponing gender transition until they secured the necessary money. As reported by an IDI participant who wished to have surgery: “The cost of [top] surgery is very high, and also different doctors used to charge different fees for it. So, to get this surgery, I first need to work and then only plan further for it.” (22, Jamshedpur, Jharkhand, HI16).

Participants from rural areas faced difficulties finding transition-related services, with some resorting to travelling or relocating to more urban areas. For example, an IDI participant from a rural area said, “I had no idea where to go or what to do. I went to a hospital [in a rural area], and they said this is a very dangerous procedure…it does not happen here. You have to go to another country. Then I went to Mumbai and started treatment there.” (25, Mumbai, MI24)

While affordability was a common barrier for all participants seeking services, it was a particular challenge for younger participants who did not have financial independence or a regular income. Lack of savings and employment made one IDI participant postpone his transition dream: “I was told that I can get it done whenever I want. However, I do not have much savings of my own, so I am not moving ahead with this. When I have good savings I will think about it, but I will get it done in the future.” (26, Chennai, Tamil Nadu, HI22)

### Weighing the quality of services

IDI participants described a wide range of reactions to their gender identities from medical staff. Negative experiences included refusal to provide transition-related care, invasive questioning, and discouragement. One participant shared how an endocrinologist discouraged him from initiating hormone therapy: “After that, I went to an endocrinologist. I chatted with him online. He said, ’Do not do this. Your family will throw you out of the house. You are not doing good’. Being an endocrinologist, he knew that these things [transitioning] can be done, but still he told me that I should not do it.” (23, Panchkula, Haryana, HI20)

Nevertheless, it is encouraging that of survey participants who had seen an endocrinologist for hormone therapy, the vast majority reported that their endocrinologist was mostly or very knowledgeable about trans health (91.0%) and that they were comfortable discussing their health care needs with the endocrinologist (89.9%). Of course, this does not preclude prior negative experiences with endocrinologists. In contrast, they reported lower levels of perceived knowledge (63.7%) and comfort (54.4%) with regular doctors.

Most interviewees reported that adequate information was provided before scheduling surgeries or initiating hormones. One participant, however, reported that his doctor only provided basic information because he did not want to “scare” him with too much information:

The doctor would tell me to ‘Do regular blood tests…full body examination. Keep eating and limit drinking. You must take care of your body more than other people’. He did not say anything else [so as] to not scare me.” (29, Nagpur, Maharashtra, MI17)

Even participants with prescriptions reported having had negative experiences when trying to get hormone injections from local clinics. An IDI participant who went to a local clinic for injection had this experience to share: “I went to a clinic there, to take HRT, testosterone injection…the behaviour of the staff was very strange. They asked me, ‘what are you doing?’ so I felt very bad. The behaviour of those people had also changed towards me…They all were looking at me weirdly. Seeing all this, I felt very bad–what these people are thinking about me?” (26, Chennai, Tamil Nadu, HI22).

A few IDI participants reported positive experiences in government hospitals. One said, “The people [counsellors, doctors] in the government hospital were explaining a lot to me. Initially, I was not a bit comfortable because it was the first time…But they treated me so well.” (28, Mumbai, MI16). However, sometimes discrimination (e.g., misgendering, misnaming) and inflexible schedules in government hospitals made participants switch to private hospitals: “I had saved money as I wanted to have surgery…we got to know both the government and the private sector. We decided to go into government as private was costly. We went to a hospital and inquired about it…After that, we went there for four to five months to get a date for the surgery. Then we got the date. But it didn’t work there. Then I saved some money and went to a private hospital” (36, Pune, Maharashtra, MI26). Such challenges accessing transition-related surgery in the public system may account for the high level of unmet need for surgery among survey participants.

Some participants who were able to access surgery in private hospitals felt that their gender identities and transition-related needs were respected. As shared by a participant who went to a private hospital that has experience in offering gender-affirmative surgeries: “They listened to me. I went to the surgeon, and they included a plastic surgeon in the team, and then they did the surgery. Their response was good.” (24, Pune, Maharashtra, MI11).

### Passing the assessment

At the time of data collection, the 7th version of the World Professional Association for Transgender Health Standards of Care recommended one mental health assessment before beginning hormone therapy or chest surgery and two assessments before genital surgery [38]. The WPATH Standards do not require that patients receive a formal diagnosis of gender dysphoria or incongruence unless needed for insurance reimbursement purposes. The Standards of Care by the Association for Transgender Health in India [12] recommend a formal diagnosis and two assessments before any surgery but were issued around the time of study data collection and were thus unlikely to have directly influenced the care participants received. Almost all IDI participants reported that the path to gender-affirmative hormone therapy or surgery began with acquiring a referral letter or psychological assessment report from one or more psychiatrists or psychologists; 67.3% of survey participants indicated they had seen a psychiatrist for such a referral. IDI participants colloquially referred to this report as a “GID certificate” even though “GID or Gender Identity Disorder” is an obsolete term (replaced with gender dysphoria in the 5^th^ edition of the Diagnostic and Statistical Manual of Mental Disorders [39].

During this process, some interviewees felt that their psychiatrist or psychologist was ‘in charge’ of their access to transition-related care and they were intimidated by the amount of power that psychiatrists had over their access to transition care. One participant reported that he could not get a report from his psychiatrist in a government hospital even after a year had passed while his peers received it within a few months from private hospitals: “In 2018, I went to a government hospital. The [psychiatrist] counselled me for almost a year. Everyone in this [support] group used to tell that it takes one or two months [to get a “GD” certificate in private hospitals] but it is not that easy to get it from a government hospital.” (23, Panchkula, Haryana, HI20)

Consequently, transmasculine community members were sometimes cautious about revealing any doubts or mental health challenges they were facing, as they worried that the psychiatrist might refuse to provide a letter. For example, one participant shared this experience:

My friends told me that whatever questions asked by the [psychiatrist], ‘Speak positively: if you feel disturbed or negative, or uncomfortable, the doctor will cancel your GID [diagnosis/certificate].’ I answered positively and gave the test. After the test, they gave me a GID [certificate]. In one day, I took two GIDs [certificates] because I had to do a small surgery. One or two GID is compulsory, so I took two.” (26, Kalyan, Maharashtra, MI12)

Notably, survey participants who had visited a psychiatrist for a “G[I]D letter” reported largely positive experiences (Table 2): 14.6% reported at least one negative experience (e.g., inappropriate or invasive questions) while 88.1% reported at least one positive experience (e.g., trans-friendly and respectful language). These positive experiences may be related to the use of peer referrals to transition-related care providers.

The high costs of getting a report from a psychiatrist prevented some participants from initiating the gender transition process. As an IDI participant said, “Two certificates are required for surgery, but I do not know how I will get that. I am saving some money for these things so that I can do all this in the future.” (26, Chennai, Tamil Nadu, HI22)

### Achieving transition goals

Although barriers to transition-related care were common, with over one-third of the survey sample waiting to begin medical transition, IDI participants who were able to access care reported reductions in gender dysphoria and increased life satisfaction. As a 24-year-old in Delhi said, “I felt great when I saw myself…That now I am changing. I am getting or am going to get what I want in life.”

Some participants were dissatisfied with their surgical results, primarily because of a lack of surgical expertise in transmasculine surgeries: “I would say that there were a few problems with the surgery, which I assume are there. I am not 100% satisfied but 80% satisfied with the surgery because there were a lot of problems.” (45, Mumbai, MI14) In line with these narratives, over one-quarter of survey participants who had surgery (26.2%) reported that they were somewhat or very dissatisfied with the results. An IDI participant emphasized, however, that despite some dissatisfaction, chest surgery boosted his confidence: “The surgery is done, I am 70% happy with it, but I got at least a flat chest. And it increased my confidence very much.” (43, Mumbai, MI13)

## Discussion

This study is the largest mixed methods study conducted with transmasculine people in India, enrolling 377 survey participants across 22 states over three months and 40 qualitative in-depth interview participants across 10 states–reflecting the strengths of a community-based participatory research project. We identified considerable challenges faced by transmasculine people in accessing and using affordable hormone therapy and surgeries. Improving access to quality and free or affordable transition-related care requires actions at multiple levels–especially from the central and state governments as mandated by the Transgender Persons (Protection of Rights) Act, 2019.

The study findings are consistent with a model of access to gender-affirmative healthcare developed in a recent systematic review and meta-ethnography of 10 qualitative studies on the experiences of transgender and non-binary youth accessing gender care [40]. The “Rainbow Brick Road” model describes the unique challenges faced by trans youth in navigating gender-affirmative healthcare and conceptualises that access requires five roads or components—gender disclosure, pursuit of care, cost of care, patient-provider dynamics, and caregiver dynamics. Although our study was not limited to youth, our participants were mostly young adults for whom ‘caregiver’ dynamics are relevant, particularly as almost half lived with their families of origin [37]. Future studies can explicitly use, adapt and expand on this model for transmasculine and gender non-binary people in India. While access to routine or general medical care was not the focus of the present study, a qualitative study conducted in 2021 [41] explored experiences of 63 transgender people (including 11 transmasculine persons) in accessing routine health care and documented the challenges faced by transmasculine persons, which included verbal and sexual abuse (by ward staff), and not being provided a doctor of preferred gender. Additional such studies that focus on transmasculine people’s access to various general and specialist medical services are needed.

Gender affirmation through medical or surgical procedures is necessary for many transgender people. While not all trans people desire those procedures, they should be made available for those who need or want them. Gender-affirmative hormone therapy (e.g., testosterone injections for transmasculine persons) is still unavailable even in most tertiary-care level public hospitals in India. This study as well as other studies from India [20, 42] have documented the challenges faced by trans people in accessing hormone therapy and surgeries despite the Transgender Persons Act explicitly directing the government to provide hormone therapy in public hospitals. To ensure easy availability of hormonal medications (e.g., testosterone injections and patches), listing those medications under the ‘National Essential Medicines List’ [43] could help improve their availability in government hospitals. Also, unlike the increasingly used ‘informed consent model’ for initiating hormone therapy [44], doctors in India still ask for at least one psychological assessment report before initiating hormone therapy. Like other studies [1], this study found that difficulties in getting hormone therapy in a timely manner from qualified healthcare professionals contributed to self-administration of hormones and getting injections over the counter and from the Internet. Brief training for trans-friendly doctors on providing transition-related hormone therapy could help improve access to safe hormone regimens.

Most study participants needed some level of transition-related medical care; however, few had completed the care they needed. Over one-third of the participants were planning to transition medically but had not begun the process; the unmet need for transition-related surgery was particularly high (69% desired to have chest surgery and 80% wanted hysterectomy or genital surgery). Interestingly, desire for genital surgery was far more common in our sample than in research from other settings; for example, over two-thirds of trans men in the United States reported not wanting or being unsure about having genital surgery [5].

Our findings revealed that most transmasculine people could not afford the high costs of surgeries in private hospitals; many worked hard to save money for surgeries or received financial support from friends or family members. Thus, access to transition-related surgeries is minimal for transmasculine people with low incomes and without family support. Cost has also been identified as a major barrier to accessing surgery for transmasculine people in the United States, with many delaying or foregoing procedures due to financial constraints [45] or resorting to crowdfunding to cover costs [46]. However, growing coverage of transition-related care by public and private U.S. health insurers has increased access to surgery in recent years [47, 48], underscoring the need to expand surgical capacity and competence within the Indian public healthcare system, as well as coverage by private insurance. The study participants reported having had primarily negative experiences in public hospitals including a lack of availability of transition-related procedures or considerable delay in undergoing surgeries. Further, dissatisfaction with surgical results was common, reflecting inadequate expertise for transmasculine surgeries in India.

A particular challenge that many study participants reported was getting a “GID certificate”, which is a psychological assessment report from a psychiatrist or psychologist, as a pre-requisite for initiation of hormones or surgeries. The WHO has removed “gender identity disorder” from the list of psychiatric disorders and introduced “gender incongruence” (mismatch between self-affirmed gender and gender assigned at birth) in the sexual health chapter of the International Classification of Diseases-11 [49]. This change should be reflected in practice, and any psychological assessment should be to support trans person’s mental health and not to make a “diagnosis” that they have a “disorder” related to their gender identity. Such a stance is consistent with the Supreme Court’s 2014 judgement that recognizes self-affirmation of gender identity without needing medical intervention [50]. Additionally, the 2014 judgement recommended that WPATH guidelines be followed until the government develops national guidelines [10]. Notably, Version 8 of the WPATH Standards of Care drops the requirement for a mental health assessment prior to hormone therapy or surgery [3, 4]. Building on global guidelines from WPATH, national and regional guidelines have been developed by professional associations in other countries [51, 52]; guidelines have been developed in India [12] but have not been endorsed by the government or adopted nationally. However, some positive developments include the 2022 approval and launch of coverage for transition-related procedures in the central government’s health insurance scheme through provision of an “Ayushman Bharat TG plus card” [53] and reimbursement of transition-related procedures by a few state governments [54], as well as the recent launch of several outpatient clinics providing transition-related care in government hospitals and community-based organizations.

In addition to taking steps to improve expertise on transition-related procedures in government hospitals, incorporating accurate information on trans people and their health needs in medical, nursing and paramedical curricula will help create a positive clinical environment for all trans people. Research on trans-inclusive medical education highlights the importance of incorporating inclusive content throughout the curriculum, skills-based education, and involvement of trans people as advisors, educators, and students [55, 56]. Education in clinical competencies is especially needed; surveys of medical students in South Asia indicate high awareness of social stigma faced by trans people but poor knowledge of trans-specific medical needs [57, 58]. In the Indian context, core competencies for trans-affirmative medical education were recently published by trans health advocates [59], which are yet to be included as part of medical education curricula.

When comparing our findings to studies focused on transfeminine persons or trans women in India, several parallels emerge. Trans women in India faced similar barriers in accessing hormone therapy, including high costs and a lack of trans-competent healthcare providers [20]. However, transfeminine individuals appeared to have greater access to peer support and community-based organizations for transition-related care, possibly due to the longer-standing visibility of hijra communities [20, 60]. Our study suggests that transmasculine people may benefit from similar community support structures, which could provide information on trans-friendly healthcare providers, financial assistance for transition-related care, and emotional support throughout the transition process.

Additionally, research on HIV service access among transgender women in India has highlighted common themes of discrimination and stigma in healthcare settings [18, 61]. While HIV-specific services have made progress in becoming more trans-inclusive, our findings indicate that transition-related care services lag in this regard. This points to the need for broader trans-competency training across all areas of healthcare, which can help reduce discrimination and stigma in healthcare settings. This aligns with findings from other low- and middle-income countries, where transgender individuals often face significant barriers in accessing both general and transition-specific healthcare due to discrimination and lack of provider knowledge [62].

This study has limitations as well as strengths. It is the first nation-wide mixed methods study of transmasculine people across India and has documented the current situation of transmasculine people’s access to and use of transition-related care. The mixed methods design helped understand both the nature and extent of the challenges faced by transmasculine people, contributing to methods and source triangulation and thereby improving the validity of the findings. Validity was further increased by engaging transmasculine communities in all stages of this study (design, data collection, and data analyses) and by having trained peer researchers who collected data. Given the use of peer-driven, non-probability sampling, findings may not be generalizable to transmasculine people across India. In addition, although Our Health Matters was open to both trans men and non-binary transmasculine people, few participants (and no IDI participants) identified as non-binary and thus this study cannot address their potentially unique experiences accessing care [63]. Although efforts were made to ensure language accessibility and sample purposively for maximum diversity across geographic location, socioeconomic status, religion, and caste, the use of virtual IDIs likely limited the representativeness of the qualitative sample. Similarly, although the quantitative survey was multi-mode, most data were collected online and in English, limiting representation of lower-income and less highly educated transmasculine persons. However, we strove to capture the considerable diversity among transmasculine people by including participants from diverse regions and states, using several languages for data collection, and offering in-person participation in select regions.

Unlike in our study, where participants primarily relied on peer networks for information, transmasculine people in high-income countries often report receiving information from healthcare providers or LGBTQ+ organizations [64]. This difference underscores the need for improved formal sources of information on transition-related care in India. Peers and grassroots organizations may play a key role in facilitating access to gender-affirming care for transmasculine individuals in India. However, we acknowledge that this study did not explore in depth the specific mechanisms by which these networks operate or their potential limitations. Future research should focus on identifying the various social and peer support systems available for transmasculine people and examining in detail how they function, their strengths and weaknesses, and their impact on healthcare access. Future studies could also collect detailed information on the experiences of transmasculine people after their transition-related surgeries, including post-operative complications, quality of post-operative care, perceived skills of surgeons, and specific aspects of dissatisfaction with surgical outcomes. Such research would be invaluable in identifying targeted improvements in surgical procedures and post-operative care for transmasculine individuals in India.

## Conclusion

We found multi-level barriers faced by transmasculine people in accessing and using transition-related health care. Given that the central and state governments are required to provide free transition-related medical care for trans people, as noted in the 2019 Transgender Persons Act, several steps need to be taken to ensure the availability of free and affordable care in at least one government hospital in each state (as noted in the Rules, 2020). These steps may include ensuring the availability of surgical expertise on transmasculine surgeries in designated government hospitals, and training to improve cultural and technical competencies of healthcare providers on trans health and to reduce discrimination. Future research should focus on assessing the quality, acceptability, and cost-effectiveness of gender-affirmative care models in public healthcare settings to assist in effective scale-up and sustainability of such models.

## Supporting information

S1 Checklist Inclusivity in global research. (DOCX)

S1 Table Additional participant quotes. (DOCX)
