# Supplementary material for: Access to transition-related health care among transmasculine people in India: A mixed-methods investigation
Source: PLOS Glob Public Health. 2024 Oct 29;4(10):e0003506. doi: 10.1371/journal.pgph.0003506 (PMC11521280; doi:10.1371/journal.pgph.0003506)
Supplement: S1 Table — (DOCX) [file pgph.0003506.s002.docx]

**Supplemental Table S1**. Additional participant quotes.

| **Theme** | **Supporting Quotes** |
| --- | --- |
| Finding information about transition-related procedures and services, p.11 | *I was scared but when they started telling their experiences, their photos started showing that this is how we used to be. Then I increased the contact with them, and a friendly atmosphere was created. That was the experience that I was initially scared of. Now I can easily find it and get to know it from the way they talk.* (28, Mumbai, MI16)  *There is a group of transgender people who used to be on WhatsApp and now on Telegram. It’s the main one and a lot of meetings were held in it. It was very informative. That's when I realized I could do surgery in three or four ways…So, it was a good experience to get a lot of information.* (23, Mumbai, MI18)  *I discovered all this on YouTube and connected with some people on Instagram. I asked them how they did it and what they did. I have also joined a counseling group in Maharashtra. Earlier, I had joined a counseling group in Hyderabad but did not get much information. The message in the group was just good morning and good afternoon. I have mostly watched YouTube to see how someone benefited from it and what the consequences were. See how someone took hormones. I didn't get as much knowledge from counseling as I did on YouTube.* (25, Maharashtra, MI15) |
| Weighing the affordability of services, p.16 | *When I looked on YouTube, I met a doctor from Delhi and then I visited him. He explained everything to me. But their rates were too high. Everything is done but how to pay lakhs of rupees. It was not affordable.* (29, Nagpur, Maharashtra, MI17)  *First, I saved from my salary and then started [medical transition]. Because if I didn't have anything, how could I start? Mainly I had a girlfriend to help me with money and more. She was supporting me in everything.* (25, Mumbai, MI24)  *I want to do it [surgery] as quickly as possible, but I don’t have any money for it. Right now, I don’t have a job. So, when I get a proper job, I will save money for it and do my top surgery.* (22, Jamshedpur, Jharkhand, HI16) |
| Weighing the quality of services, p.19 | *So, I went to the doctor to get an injection, and he asked if I had surgery. So, it was a little uncomfortable. If a person is transitioning, it is not good to ask him directly*. (23, Mumbai, MI18, Marathi)  *The doctors where I went for a blood test are very friendly. He [the doctor] was sharing his experience with some trans men staying near to him. This was not new to him… I also told him that I have a lot of friends who don't feel like going out, they feel very ashamed. If you are very friendly, I will bring them here. So, he said ‘yes, bring them without hesitation.’* (28, Mumbai, MI16)  *But about bottom surgery, I am not sure. I don’t think I need my uterus removal surgery. If needed, I can go for hysto [hysterectomy]; otherwise, I don’t want to. As per my knowledge, bottom surgery is costly, and the success rate is low. That’s why I am scared to go for bottom surgery.* (23, Lucknow, Uttar Pradesh, HI23) |
| *Passing the assessment, p.20* | *I met the doctor, he already gave certificates to lots of trans man. He is the very intelligent doctor for this. So I feel very happy, he gave me a great response. He is also very trans-friendly. He asked me some questions and then gave GID*. (23, Churu, Rajasthan, HI11)  *The doctor who gave me this certificate was very good and communicated well with me… I have heard from many that their psychiatrist called them with the wrong gender or name, asked weird questions about their gender, and mentioned the wrong name or gender on their gender dysphoria certificate. But in my case, I didn’t have to face anything like that. My psychiatrist addressed me with the correct name and gender and didn’t ask any weird questions regarding my gender*. (23, Uttar Pradesh, HI23)  *Before GID he counseled me. I had a lot of tests. There were four hundred + questions that were asked in turn. He then asked for information about my family, what I do, whether I’m in a relationship? I mean, they tried to know all my information first, tested my IQ. It contains a piece of paper, and the colors are left in it. They ask what you see in it and so on…So, I was a little scared whether I was like that or not. Whether I am going to get GID or not? I had a lot of obstacles. I was getting tired. The chief doctor said ‘He is a boy. There is no need to look at all this. Give him the same certificate.’ Then I got the GID.* (28, Mumbai, MI16) |
| *Achieving transition goals, p.22* | *Transition plays a big part in this. That is why I have become so strong mentally. I got what I wanted. I am living the life I used to think about. So that thing is very important to improve my mental health.* (23, Mumbai, MH25)  *Because before, even though I wore clothes or binders to hide my body features, people still doubted me by looking closely at my face, but now, after taking HRT, this problem has decreased a lot. Not everything is good, but I still believe the bad comments after HRT have decreased slightly. There’s a huge change in terms of safety.* (23, Lucknow, Uttar Pradesh, HI23)  *I didn’t even look in the mirror until I had my upper surgery... I have satisfaction after the surgery that I now look in the mirror at what I think of myself. So, I think the transition is important.* (23, Mumbai, MI18) |
